# Stability of the Distribution of Patient Health Questionnaire-9 Scores Against Age in the General Population: Data From the National Health and Nutrition Examination Survey

**DOI:** 10.3389/fpsyt.2018.00390

**Published:** 2018-08-23

**Authors:** Shinichiro Tomitaka, Yohei Kawasaki, Kazuki Ide, Maiko Akutagawa, Yutaka Ono, Toshiaki A. Furukawa

**Affiliations:** ^1^Department of Mental Health Panasonic Health Center, Tokyo, Japan; ^2^Department of Health Promotion and Human Behavior, Kyoto University Graduate School of Medicine School of Public Health, Kyoto, Japan; ^3^Clinical Research Center, Chiba University Hospital, Chiba, Japan; ^4^Department of Pharmacoepidemiology, Graduate School of Medicine and Public Health, Kyoto University, Kyoto, Japan; ^5^Center for the Promotion of Interdisciplinary Education and Research Kyoto University, Kyoto, Japan; ^6^Department of Drug Evaluation and Informatics, School of Pharmaceutical Sciences, University of Shizuoka, Shizuoka, Japan; ^7^Center for the Development of Cognitive Behavior Therapy Training, Tokyo, Japan

**Keywords:** depressive symptoms, Patient Health Questionnaire-9, age, National Health and Nutrition Examination Survey, exponential distribution, overlap coefficients

## Abstract

**Background:** Epidemiological studies using the nine-item Patient Health Questionnaire (PHQ-9) have reported inconsistencies regarding the relationship between age and total scores. To determine whether this discrepancy is due to the stability of the distribution of PHQ-9 total scores against age, we investigated whether the total score distribution remains stable during adulthood, and also investigated the mathematical patterns of the total score distribution.

**Methods:** The present study utilized data from 15,847 participants of the 2009–2014 United States National Health and Nutrition Examination Survey, all of whom responded to all PHQ-9 items. The stability of the total score distribution among different age groups was examined using overlap coefficients and graphical analysis.

**Results:** High overlap coefficients were observed between all age groups for the distributions of PHQ-9 total scores, suggesting that the distribution of PHQ-9 total scores remains stable against age. Graphical analysis demonstrated that distributions of PHQ-9 total scores were similar across age groups. In addition, distributions of PHQ-9 total scores exhibited an exponential pattern, except at the lower end of the distribution.

**Conclusions:** Our findings indicate that the stability of the distribution of PHQ-9 total scores throughout adulthood may underlie inconsistencies in the evidence regarding age-related changes in total depression scores.

## Introduction

Clinical and scientific research studies have aimed to identify the age at which people become more susceptible to depression (1). Because the diagnosis of depression is based on the degree of depressive symptoms, there has been much interest in understanding the relationship between age and depressive symptoms in the general population (2–4). Numerous epidemiological studies have attempted to clarify age-related changes in depressive symptoms (1, 3, 5–7). However, epidemiological studies have reported inconsistent evidence regarding age-related changes in total scores on depression screening scales during adulthood, suggesting a difficulty in replicating the relationship between age and depressive symptoms using traditional screening instruments.

The Center for Epidemiologic Studies Depression Scale (CES-D), developed by Radloff in 1977, is a self-reported depressive symptoms questionnaire that now serves as a screening tool for depression in primary care and research settings (2). Although some studies have reported inconsistent results (1), several cross-sectional surveys and longitudinal studies—the majority of which utilized the CES-D—have demonstrated that the trajectory of depressive symptom scores follows a U-shaped pattern, with total scores being high during young adulthood, decreasing during middle adulthood, and then increasing again after the age of 70 (5, 6, 8, 9).

The nine-item Patient Health Questionnaire (PHQ-9), developed in the 1990s (10), consists of questions associated with the nine criteria for depression outlined in the Diagnostic and Statistical Manual of Mental Disorders (11), and is widely used for the self-rating of depression worldwide (12). Kroenke et al. reported that a PHQ-9 score ≥10 had 88% sensitivity and 88% specificity for major depression in an assessment of criterion validity (13). Several lines of evidence suggest that the PHQ-9 total score distributions are right-skewed in the general population and a PHQ-9 score of 5 approximately corresponds to a percentile rank of 80% and, respectively, of 10 to a rank of 95%, and of 15 to a rank of 99% (14, 15).

Of note, epidemiological studies using the PHQ-9 and its related versions have reported greater inconsistency in age-related changes in depressive symptoms than those using the CES-D (5, 6, 14–18). Contrary to the results obtained using the CES-D, the National Health and Nutrition Examination Survey (NHANES) and the Behavioral Risk Factor Surveillance Survey (BRFSS) in the United States reported that the trajectory of symptom changes followed a reverse U-shaped pattern, with total scores being low during young adulthood, increasing during middle adulthood, and then decreasing again after the age of 60 (16, 19). In contrast, three German surveys and an Indian survey reported that total PHQ-9 scores increased with age (14, 15, 17, 20), while a population-based study in China reported that total scores decreased with age (18). Furthermore, epidemiological studies using other depression screening scales, such as the Hospital Anxiety and Depression Scale (HADS), have reported trajectories that differ from those obtained using the CES-D (21, 22).

Although the reason for the difficulty in replicating the relationship between age and total depression scores remains unclear, one possibility is that the effect of age on total scores using such a scale is so small that it is difficult to reproduce the association between age and total scores. Generally, it is easy to demonstrate large effects. Conversely, it is difficult to replicate small effects because they are easily neutralized by other factors (23). Epidemiological studies have reported that sex, ethnicity, education level, employment status, marital status, and household income significantly influence depressive symptoms (17, 19, 24). In addition, most of these factors differ according to age group in population-based studies. For example, there is an increasing amount of racial and ethnic diversity among young age groups in industrialized nations. Thus, if the effect of age on the PHQ-9 total scores is small, and the other factors differ according to age group, it will be difficult to reproduce the relationship between age and PHQ-9 total scores.

**Figure 1:**
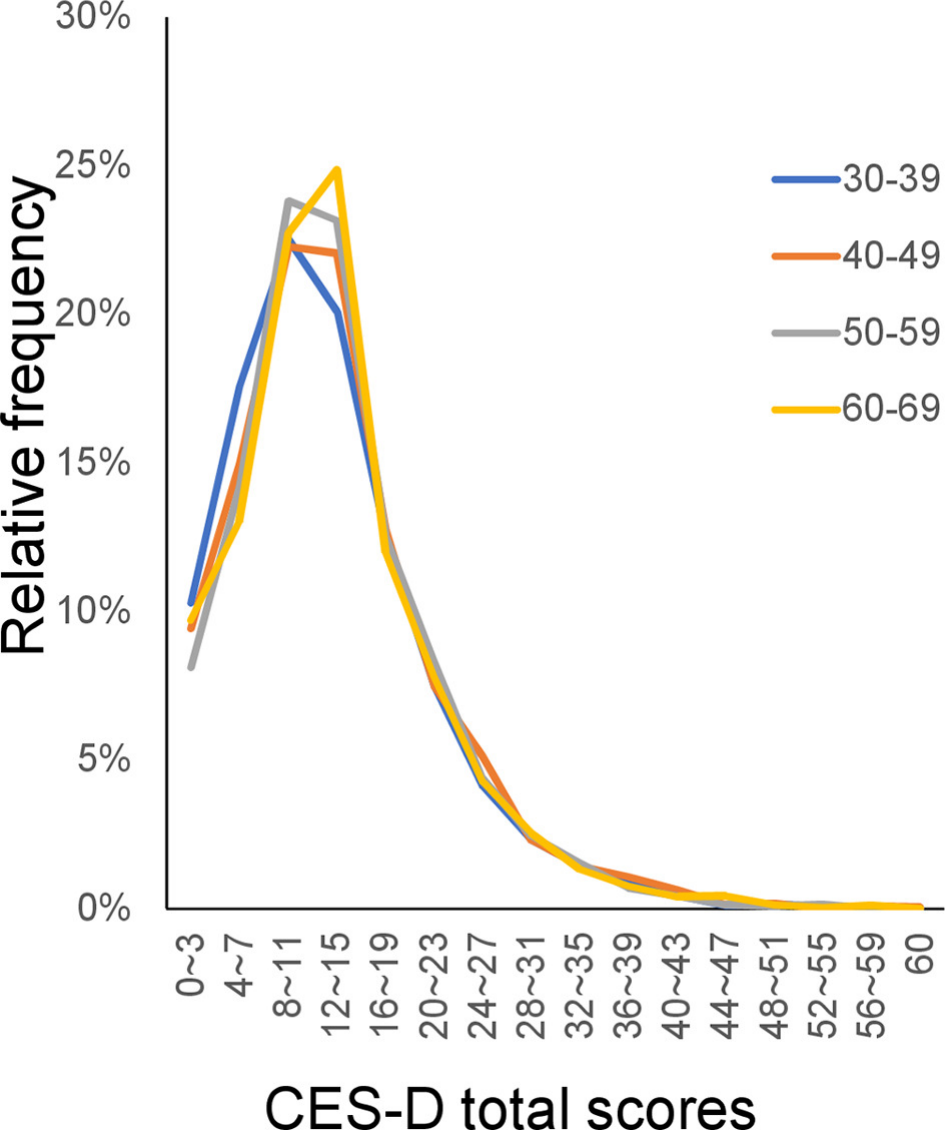
CES-D distributions for the middle adulthood group. The distributions of CES-D scores were similar among the middle adulthood group. (https://doi.org/10.1371/journal.pone.0114624). CES-D, Center for Epidemiologic Studies Depression Scale.

Consistent with this hypothesis, although cross-sectional surveys using other depression screening scales have reported inconsistent trajectories of depressive symptom scores, the reported age-related changes in depressive symptoms have been relatively mild (5, 9, 21, 22). Furthermore, in one previous study, the distribution of total scores on the CES-D was stable during middle adulthood, during which time no significant differences in mean total scores were observed (Figure 1) (9). These results suggest that the stability of the total score distribution for depression screening scales against age underlies inconsistencies in the evidence regarding the relationship between age and total depression scores. To test the hypothesis that inconsistencies in the evidence regarding the relationship between age and total depression scores is due to the stability of the distribution of PHQ-9 total scores against age, it is first necessary to ascertain the similarities in the distribution of PHQ-9 total scores throughout adulthood. Of note, our hypothesis is based on the degree of similarity rather than the equality of the distributions.

Previous studies have reported that total PHQ-9 scores in the general population follow an exponential distribution, except at the lower end of the distribution (25). This exponential pattern has been observed in previous studies that have utilized other scales, such as the CES-D, Kessler Psychological Distress Scale (K6), and Clinical Interview Schedule-Revised (CIS-R) (9, 26, 27). In general, an exponential distribution emerges when the total stability of the distribution is observed in conjunction with individual variability in the indicated variables (28, 29), suggesting that the exponential pattern of the distribution is linked to the stability of the total score distribution.

The present study investigated whether the distribution of total scores on the PHQ-9 remains stable across all age groups in the general population. The degree of similarity for total score distributions among age groups was quantified using overlap coefficients. Furthermore, we used graphical analysis to evaluate the similarity of the distributions among age groups (30) and examined whether the distribution of total PHQ-9 scores followed the same exponential pattern.

We used data from the NHANES—a national survey designed to assess the health and nutritional status of adults and children in the United States (31) that includes the PHQ-9 (12). The PHQ-9 data from the NHANES are suitable for verifying the aforementioned assumption due to the large sample sizes and limited selection bias.

## Methods

### Dataset

We analyzed data from the 2009–2014 NHANES. The NHANES includes a nationally representative sample of non-institutionalized civilian US citizens selected using a multiple-stage design. The survey consisted of a household interview and an examination conducted in a mobile examination center. All survey participants provided written informed consent. De-identified data from the NHANES are available for researchers worldwide (32). For the 2009–2014 NHANES, 41,035 participants were selected. The sociodemographic characteristics of the 2009–2014 NHANES samples are reported in detail elsewhere (32). Among all participants selected, approximately half were selected to respond to the PHQ-9. We used data from 17,899 participants aged 18 and older. Since our local institutional review board does not regard de-identified public data analysis as human subjects research, our research did not require ethical approval from the board.

### Measures

In the 2009–2014 NHANES, depressive symptoms were assessed using the PHQ-9. Respondents self-rated the frequency of a variety of depressive symptoms within the past 2 weeks along a 4-point scale, as follows: 0 = “not at all,” 1 = “several days,” 2 = “more than half the days,” and 3 = “nearly every day.” Total scores ranged from 0 to 27.

### Analysis

Participants were categorized into the following age groups: 18–19, 20–29, 30–39, 40–49, 50–59, 60–69, 70–79, and 80 years and older. Descriptive statistics (e.g., mean, standard deviation, skewness, kurtosis, and frequency curve) were calculated for each age group. To estimate the proportion of high PHQ-9 total scores, we calculated the 90th percentile of the PHQ-9 total score for each age group. The Kruskal–Wallis test was used to analyze differences among the age groups.

**Figure 2:**
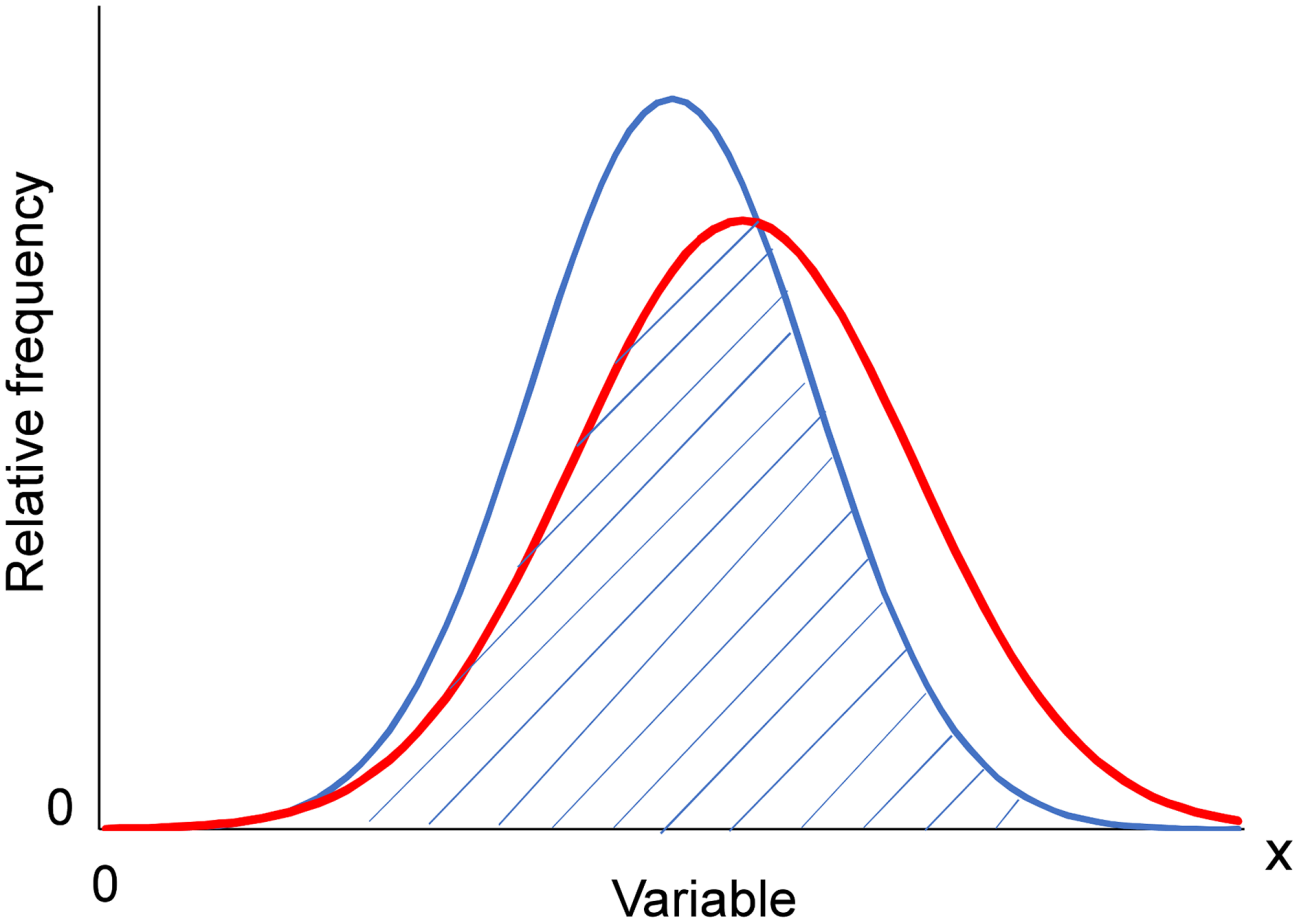
Overlap coefficient from two normal distributions. The overlap coefficient (shaded area) from two normal distributions with unequal average and variance.

To evaluate the similarity of the distributions between different age groups, we calculated the overlap coefficient of the distributions (33). Overlap coefficients represent the proportion of overlap between two probability distributions (Figure 2) and serve as a measure of the similarity between observed distributions (33). Recently, researchers have used effect sizes such as Cohen's *d* to quantify the degree of difference between two groups (34). Estimating the degree of difference based on effect sizes may serve as an alternative to similarity indices. However, effect sizes focus on the difference between a representative value (mean or median), rather than the pattern or the distribution itself. Thus, we used overlap coefficients to assess the similarities among distributions. Overlap coefficients range between zero and one.

Furthermore, we used graphical analysis to confirm the similarity of the distribution patterns between different age groups. Graphical analysis is advantageous in that it enables one to observe patterns in the data. Indeed, such analyses allowed us to detect common distribution patterns among different age groups. We used both normal and log-normal scales, the latter of which enables one to identify the range of an exponential pattern. The regression curve for an exponential model was estimated for each age group. All analyses were conducted using JMP Version 11 for Windows (SAS Institute, Inc., Cary, NC, USA).

## Results

### Analysis of PHQ-9 total scores

As we analyzed the distribution of total scores, participants who did not respond to all PHQ-9 items (2,052 individuals) were excluded from the analysis. The final sample for the analysis consisted of 15,842 individuals. The characteristics of the final sample and descriptive statistics for the distributions of PHQ-9 total scores according to age are shown in Table 1. Mean and median values were highly similar among age groups. Of note, the skewness and kurtosis values for all age groups were close to 2 and 5, respectively. It should be noted that the skewness and kurtosis of any exponential distributions are 2 and 6, and that those of any normal distributions are 0 and 0, respectively.

**Table 1:** Participant characteristics.

**Age group**	**Number**	**Female (%)**	**Mean ± S.D**.	**Skewness**	**Kurtosis**	**Median**	**90th percentile**
18–19	869	49.1	3.2 ± 3.7	2.0	4.9	2	8
20–29	2,584	49.9	3.2 ± 4.0	2.0	4.8	2	8
30–39	2,472	48.9	3.0 ± 4.3	2.1	5.2	2	9
40–49	2,551	52.4	3.5 ± 4.7	2.0	4.2	2	10
50–59	2,429	50.4	3.7 ± 5.1	1.8	3.2	2	11
60–69	2,545	50.7	3.4 ± 4.6	1.9	3.7	1	10
70–79	1,519	51.3	2.7 ± 3.8	2.2	5.8	1	8
80+	878	52.4	2.7 ± 3.7	2.1	5.0	1	7

As depicted in Figure 2, the mean values of the PHQ-9 total scores exhibited a reverse U-shaped pattern, being low during young adulthood, increasing during middle adulthood, and then decreasing during older adulthood, consistent with the findings of a previous study using NHANES data (16). Kruskal–Wallis analysis revealed that PHQ-9 total scores differed significantly among the age groups (χ^2^ = 61.8425, *P* < 0.0001).

### Overlap coefficients among different age groups

**Figure 3:**
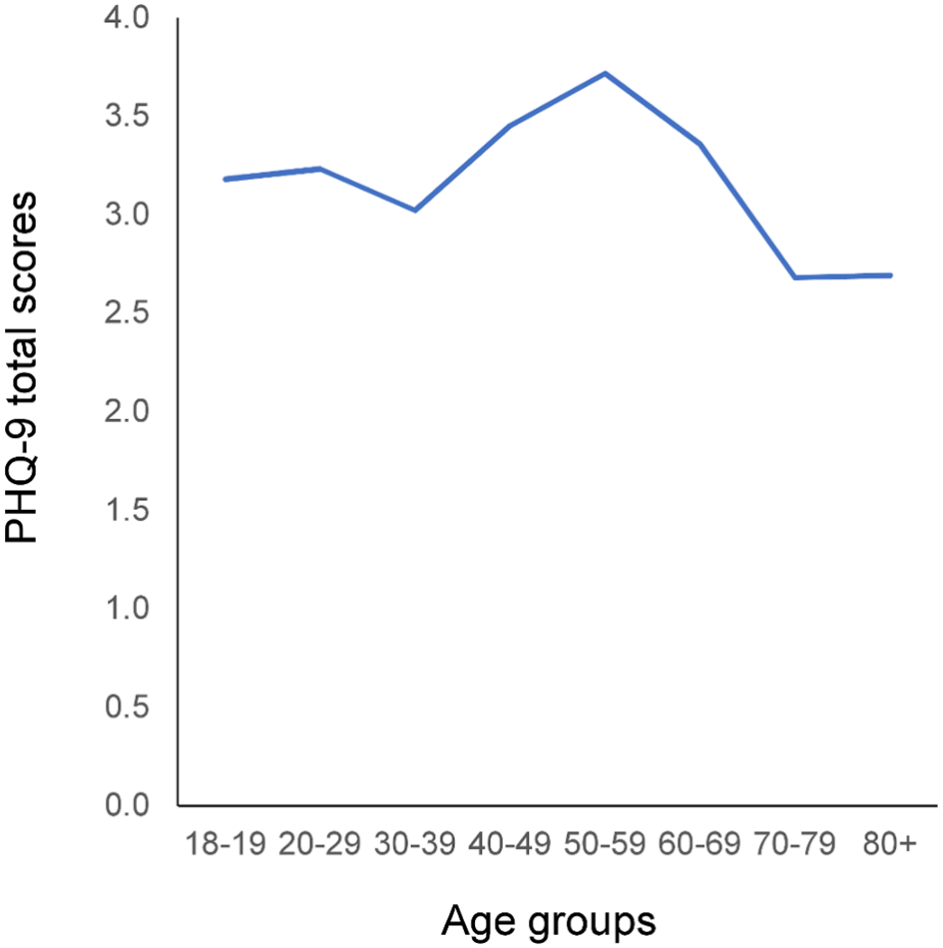
Relationship between age and mean PHQ-9 total score. Mean PHQ-9 total scores exhibited a reverse U-shaped pattern: Mean scores were low during young adulthood, increased during middle adulthood, and then decreased again during old age. PHQ-9, Patient Health Questionnaire-9.

The overlap coefficient between different age groups ranged from 0.84 (between 18–19 and 50s) to 0.96 (between 50 and 60s), demonstrating the general similarity of the distribution patterns among different age groups. The overlap coefficient between adjacent age groups ranged from 0.93 to 0.96, indicating that distributions for adjacent age groups were highly similar. Of note, the overlap coefficient among middle and older adult groups (30, 40, 50, 60, 70s, and 80+) was over 0.9 for any combination, whereas that between young adult groups (18–19 and 20s) and middle/older adult groups (30, 40, 50, 60, 70s, and 80+) was approximately less than 0.9, indicating that the distribution of PHQ-9 total scores changed more between young and middle adulthood than between middle adulthood and old age. This seemingly contradicts the finding that mean PHQ-9 total scores changed more between the 50s and 80+ groups than between the 18–19 and 50s groups (Figure 3).

### Graphical analysis of the distributions

**Figure 4:**
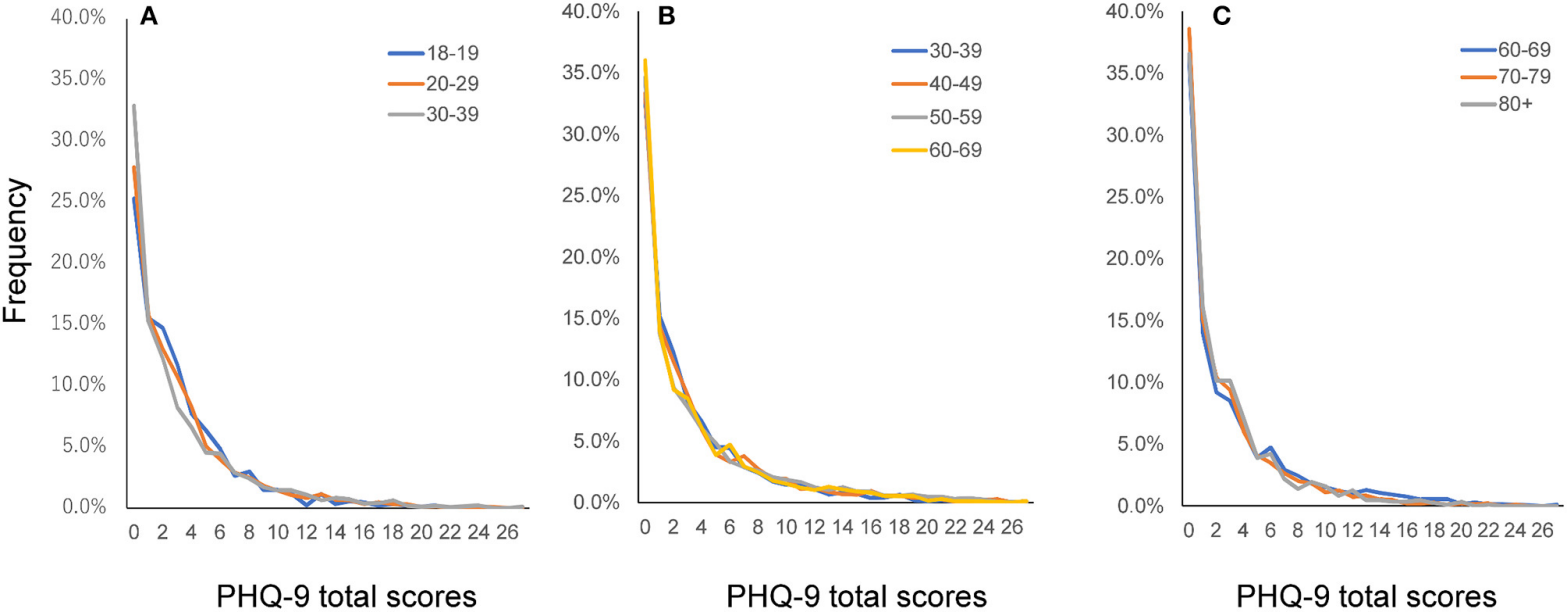
Distributions of PHQ-9 total scores according to age group. Distributions of PHQ-9 total scores for the young adulthood group **(A)**, middle adulthood group **(B)**, and old age group **(C)**. The distributions of PHQ-9 total scores were similar among all age groups. PHQ-9, Patient Health Questionnaire-9.

The distributions of the PHQ-9 total scores were compared among the young age group (18–19, 20, and 30s), the middle age group (30, 40, 50, and 60s), and the older age group (60, 70s, and 80+) (Figure 4). The distributions of the PHQ-9 total scores were commonly right-skewed and similar among all age groups. Although the mean of the PHQ-9 total scores appeared to change throughout adulthood (Figure 3), it was difficult to distinguish each age group graphically (Figure 4). These findings were in accordance with the fact that the overlap coefficient between adjacent age groups remained high throughout adulthood (Table 2). The frequencies of the zero score were 25.3, 27.9, 32.9, 33.3, 34.6, 36.0, 38.6, and 36.6% for the 18–19, 20–29, 30–39, 40–49, 50–59, 60–69, 70–79, and 80+ years groups, respectively. These results indicate that the frequencies of the zero score increased with age.

**Table 2:** Overlap coefficients among different age groups.

	**18–19**	**20–29**	**30–39**	**40–39**	**50–59**	**60–69**	**70–79**	**80+**
18–19	–	**0.94**	0.89	0.86	0.84	0.85	0.85	0.86
20–29		–	**0.93**	**0.91**	0.88	0.88	0.89	**0.90**
30–39			–	**0.95**	**0.93**	**0.94**	**0.93**	**0.92**
40–49				–	**0.95**	**0.94**	**0.93**	**0.92**
50–59					–	**0.96**	**0.92**	**0.91**
60–69						–	**0.94**	**0.93**
70–79							–	**0.95**
80+								-

*Overlap coefficients >0.9 are presented in bold*.

**Figure 5:**
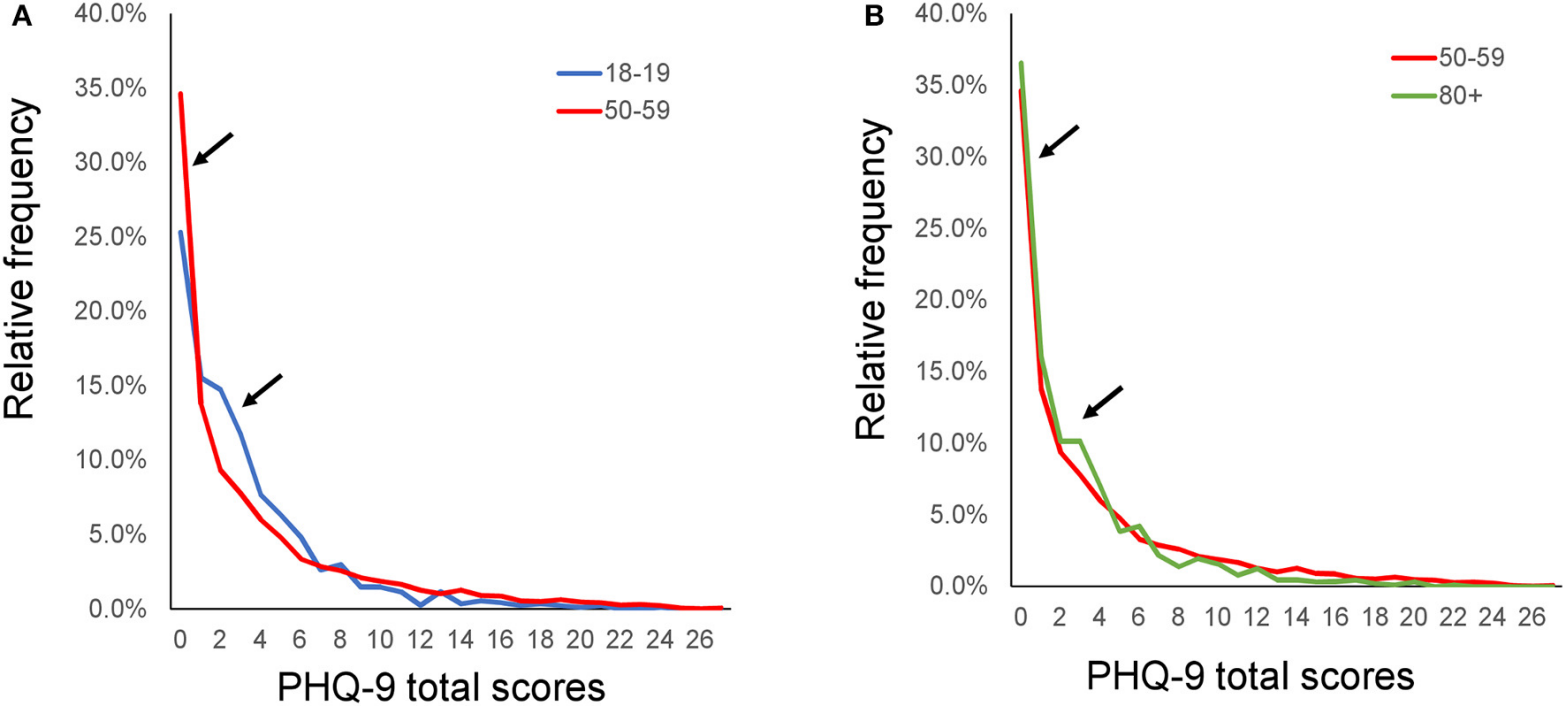
Distributions of the PHQ-9 total scores between 18–19 and 50s, and between 50s and 80+. The distributions of the PHQ-9 total scores were compared between the 18–19 and 50s groups **(A)**, and between the 50s and 80+ groups **(B)**. As indicated by the black arrows, from 0 points to 6 points, the distributions of the PHQ-9 total scores for the 18–19 and 50s groups were less similar to one another than those of the 50s and 80+ groups.

Although mean PHQ-9 total scores changed more between the 50s and 80+ groups than between the 18–19 and 50s groups (Figure 3), the overlap coefficient between the 50s and 80+ groups (0.91) was higher than that between the 18–19 and 50s groups (0.84). To clarify this discrepancy, the distributions of the PHQ-9 total scores were compared between 18–19 and 50s groups, and between the 50s and 80+ groups (Figure 5). As indicated by the black arrows, from 0 points to 6 points, the distributions of the total PHQ-9 scores for the 18–19 and 50s groups (Figure 5A) were less similar to one another than those of the 50s and 80+ groups (Figure 5B), supporting the finding that the overlap coefficient between the 18–19 and 50s groups was lower than that between the 50s and 80+ groups. As shown in Figure 5A, the relative frequencies of ages 18–19 were lower at 0 points, higher from 2 points to 6 points, and lower again from 9 points to 16 points than those of ages 50–59. These findings indicated that the change in the distribution occurring between the 18–19 and 50s age groups was balanced to the right and left, with the mean value (3.2) at the center, resulting in a relatively small difference in mean total PHQ-9 scores between the two groups.

**Figure 6:**
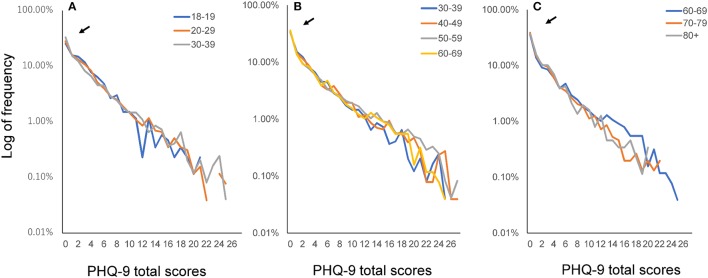
Distributions of PHQ-9 total scores according to age group using a log-normal scale. Distributions of PHQ-9 total scores for the young adulthood group **(A)**, middle adulthood group **(B)**, and old age group **(C)** on a log-normal scale. The distributions for all age groups exhibited a linear pattern on a log-normal scale. However, for PHQ-9 scores over 10 points, the curves for each age group fluctuated randomly. As indicated by the arrows, all groups exhibited higher frequencies at the zero score than those predicted based on the linear log-normal pattern. PHQ-9, Patient Health Questionnaire-9.

To demonstrate the pattern of the PHQ-9 total score distribution, we evaluated the distributions using a log-normal scale (Figure 6). The distributions for all age groups exhibited a linear pattern on a log-normal scale. However, for PHQ-9 scores over 10 points, the curves for each age group fluctuated randomly, reflecting the small sample sizes in each age group. In fact, the percentage of PHQ-9 total scores over 10 points was less than 1%. As indicated by the arrows in Figure 6, all groups exhibited higher frequencies at the zero score than those predicted based on the linear log-normal pattern. The divergence of the actual data from the predicted linear pattern at the zero score increased with increasing age.

### Regression curves for the exponential model

The fitting curves for the exponential model were calculated for the 18–19 years (y = 0.2438e^−0.25x^, *R*^2^ = 0.93), 20–29 years (y = 0.2391e^−0.24x^, *R*^2^ = 0.98), 30–39 years (y = 0.1979e^−0.22x^, *R*^2^ = 0.95), 40–49 years (y = 0.174e^−0.19x^, *R*^2^ = 0.93), 50–59 years (y = 0.16e^−0.18x^, *R*^2^ = 0.95), 60–69 years (y = 0.1974e^−0.21x^, *R*^2^ = 0.96), 70–79 (y = 0.2141e^−0.24x^, *R*^2^ = 0.96), and 80+ years groups (y = 0.2233e^−0.25x^, *R*^2^ = 0.94). In accordance with the findings obtained using the log-normal scale, higher coefficients of determination for the exponential model were observed among all age groups.

## Discussion

The present study aimed to evaluate the hypothesis that evidence regarding age-related changes in total depression scores on screening scales is inconsistent due to the stability of the total score distribution against age. Our findings indicated that the distribution of PHQ-9 total scores was generally stable throughout all periods of adulthood. These findings demonstrate the stability of the PHQ-9 total score distribution against age, suggesting a small effect of age on the distribution and supporting our hypothesis.

In the present study, the mean values of the PHQ-9 total scores exhibited a reverse U-shaped pattern during adulthood, consistent with the findings of a previous study using NHANES data (16). Although the PHQ-9 total score was significantly different among the age groups in the present study, this may be attributable to the large sample size (15,842 individuals). If a sample size is over 10,000, a significant difference is likely to be found even when the difference between groups is negligible (34). In addition, as our hypothesis was based on the similarity rather than the equality of the distributions, the identification of significant differences with large sample sizes does not contradict the hypothesis.

It remains unclear why the evidence regarding age-related changes in total scores appears to be more inconsistent for the PHQ-9 than the CES-D. However, our findings indicated that the distribution of PHQ-9 total scores was generally stable throughout adulthood. These results suggest that the stability of the distribution of the PHQ-9 total scores throughout adulthood is related to inconsistencies in evidence regarding age-related changes in total scores. Further research is required to clarify why the distribution of total scores obtained using the PHQ-9 is stable throughout a wide age range.

Overlap coefficient and graphical analysis revealed that the distribution of PHQ-9 total scores was more stable during middle adulthood and old age than during young adulthood. This result is consistent with the previous finding that the distribution of CES-D total scores was more stable during middle adulthood than during young adulthood (9). These findings suggest that, although the total score distribution on depression screening scales is generally stable throughout out all periods of adulthood, the stability of the distribution increases during middle adulthood. Of note, our results demonstrate that differences in the mean values do not necessarily correspond to differences among the distributions themselves. In fact, although mean PHQ-9 total scores differed more significantly between the 50s and 80+ groups than between the 18–19 and 50s groups, the distributions of PHQ-9 total scores were more similar to each other for the 50s and 80+ groups than for the 18–19 and 50s groups. These findings suggest that overlap coefficients and graphical analysis are adequate for evaluating the magnitude of the similarity of the distributions between groups.

In general, age has a strong impact on biological indices, such as cardiopulmonary function, exercise capacity, and brain function (35). While these indices remain stable against age in some individuals, they fluctuate in others, altering the distribution of the variables with age. To the best of our knowledge, there are few physiological variables for which it is difficult to distinguish between the distributions of individuals in their 30 vs. 60s. Conversely, although each individual's depressive symptoms often change throughout life, the distribution of PHQ-9 total scores remains stable against age. Moreover, in the previous study, the distribution of CES-D total scores was stable against age between the age of 30 and 70 years old (9). These findings could be regarded as a unique feature of depressive symptom scores. The degree to which these findings can be generalized to other depression screening scales is unknown but warrants further investigation.

In the present study, the distribution of PHQ-9 total scores followed an exponential distribution, except at the lower end of the distribution, consistent with the findings of previous studies using the CES-D, K6, and CIS-R (9, 26, 27). The exponential pattern appears to coincide with the stability of the distribution. In general, an exponential distribution arises when total stability and individual variability are observed together (i.e., maximum entropy) (28, 29). Although the mechanism underlying this exponential pattern remains unclear, our recent simulation study has demonstrated that, if the latent trait of depressive symptoms follows an exponential distribution, the total scores of a depression rating scale will follow an exponential pattern, except at the lower end of the distribution (36).

The present study possesses some limitations of note. In this study, we evaluated the similarities among distributions using overlap coefficients and graphical analysis. However, one major limitation of these methods is the lack of a unified approach to the interpretation of results. Thus, even after obtaining the results of these analyses, we were unable to describe the degree of similarity using unified descriptors, such as “small,” “medium,” “large,” etc. (37). Further research is required to develop unified descriptors for the interpretation of these results.

Furthermore, although the age at which individuals become more susceptible to depression remains an important clinical question, evidence regarding age-related changes in total depression scores during adulthood remains inconsistent due to the stability of the total score distribution against age. Thus, it may be more appropriate to focus on differences in socioeconomic status in each age group. While our results suggest that the stability of the total score distribution against age during adulthood can be generalized to other depression screening scales, further study is required to verify this hypothesis.

## Availability of data and materials

The data that support the findings of this study are available in the NHANES repository, https://www.cdc.gov/nchs/nhanes/index.htm.

## Author contributions

ST made substantial contributions to conception and design, acquisition of data, and analysis and interpretation of data and was a major contributor in writing the manuscript. YK, KI, and MA were involved in the interpretation of the data and in revising the manuscript. YO and TF were involved in the study design and revising the manuscript critically. All authors read and approved the final manuscript.

### Conflict of interest statement

The authors declare that the research was conducted in the absence of any commercial or financial relationships that could be construed as a potential conflict of interest.
